# Supplementary figures and images for: Kinetochore-independent mechanisms of sister chromosome separation
Source: PLoS Genet. 2021 Jan 29;17(1):e1009304. doi: 10.1371/journal.pgen.1009304 (PMC7886193; doi:10.1371/journal.pgen.1009304)

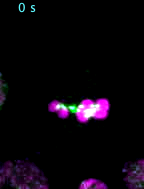

Supplement: S1 Data — Chromosomes are labeled with H2Av histone variant tagged with RFP (magenta) and cohesin is labeled with Rad21 tagged with GFP (green). Time lapse: 5 seconds. 7 frames per second. Scale bar: 2 μm. This movie corresponds to Fig 2B. (TIF) [file pgen.1009304.s003.tif]

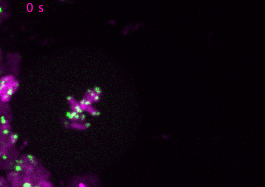

Supplement: S2 Data — Chromosomes are labeled with H2Av histone variant tagged with RFP (magenta) and telomeres are labeled with HOAP tagged with GFP (green). Time lapse: 18 seconds. 7 frames per second. Scale bar: 2 μm. This movie corresponds to Fig 3A. (TIF) [file pgen.1009304.s004.tif]

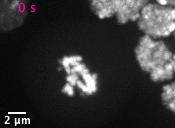

Supplement: S3 Data — Chromosomes are labeled with H2Av histone variant tagged with RFP (white). Time lapse: 8 seconds. 7 frames per second. Scale bar: 2 μm. This movie corresponds to Fig 4B. (TIF) [file pgen.1009304.s005.tif]

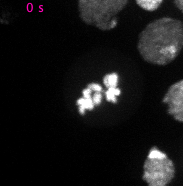

Supplement: S4 Data — Chromosomes are labeled with H2Av histone variant tagged with RFP (white). Time lapse: 10 seconds. 7 frames per second. Scale bar: 2 μm. This movie corresponds to Fig 6B. (TIF) [file pgen.1009304.s006.tif]

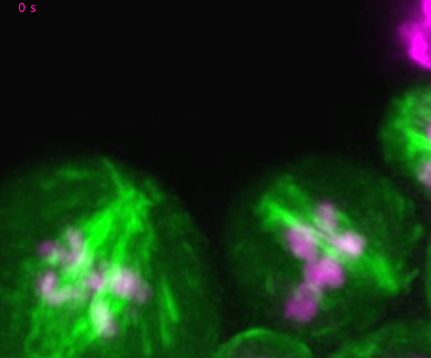

Supplement: S5 Data — Chromosomes are labeled with H2Av histone variant tagged with RFP (magenta) and EB1 is labeled with EB1 tagged with GFP (green). Time lapse: 5 seconds. 7 frames per second. Scale bar: 2 μm. This movie corresponds to Fig 7B. (TIF) [file pgen.1009304.s007.tif]
